# Benign and Deleterious Cystic Fibrosis Transmembrane Conductance Regulator Mutations Identified by Sequencing in Positive Cystic Fibrosis Newborn Screen Children from California

**DOI:** 10.1371/journal.pone.0155624

**Published:** 2016-05-23

**Authors:** Danieli B. Salinas, Patrick R. Sosnay, Colleen Azen, Suzanne Young, Karen S. Raraigh, Thomas G. Keens, Martin Kharrazi

**Affiliations:** 1 Department of Pediatrics, Division of Pediatric Pulmonology, Children’s Hospital Los Angeles, Keck School of Medicine, University of Southern California, Los Angeles, California, United States of America; 2 Department of Medicine, Division of Pulmonary and Critical Care Medicine and McKusick-Nathans Institute of Medical Genetics, Johns Hopkins University School of Medicine, Johns Hopkins University, Baltimore, Maryland, United States of America; 3 Department of Biostatistics, Southern California Clinical and Translational Science Institute, Children’s Hospital Los Angeles, Keck School of Medicine, University of Southern California, Los Angeles, California, United States of America; 4 The Sequoia Foundation, La Jolla, California, United States of America; 5 McKusick-Nathans Institute of Medical Genetics, Johns Hopkins University School of Medicine, Johns Hopkins University, Baltimore, Maryland, United States of America; 6 Division of Environmental and Occupational Disease Control, Environmental Health Investigations Branch, Environmental Epidemiology Section, California Department of Public Health, Richmond, California, United States of America; International Centre for Genetic Engineering and Biotechnology, ITALY

## Abstract

**Background:**

Of the 2007 Cystic Fibrosis Transmembrane Conductance Regulator (*CFTR)* mutations, 202 have been assigned disease liability. California’s racially diverse population, along with *CFTR* sequencing as part of newborn screening model, provides the opportunity to examine the phenotypes of children with uncategorized mutations to help inform disease liability and penetrance.

**Methods:**

We conducted a retrospective cohort study based on children screened from 2007 to 2011 and followed for two to six years. Newborns that screened positive were divided into three genotype groups: those with two CF-causing mutations (CF-C); those with one mutation of varying clinic consequence (VCC); and those with one mutation of unknown disease liability (Unknown). Sweat chloride tests, pancreatic sufficiency status, and *Pseudomonas aeruginosa* colonization were compared.

**Results:**

Children with two CF-causing mutations had a classical CF phenotype, while 5% of VCC (4/78) and 11% of Unknown (27/244) met diagnostic criteria of CF. Children carrying Unknown mutations 2215insG with D836Y, and T1036N had early and classical CF phenotype, while others carrying 1525-42G>A, L320V, L967S, R170H, and 296+28A>G had a benign clinical presentation, suggesting that these are non-CF causing.

**Conclusions:**

While most infants with VCC and Unknown *CFTR* mutations do not meet diagnostic criteria for CF, a small proportion do. These findings highlight the range of genotypes and phenotypes in the first few years of life following CF newborn screening when *CFTR* sequencing is performed.

## Introduction

Newborn screening (NBS) for cystic fibrosis (CF) is performed throughout the U.S. and other countries based on known long-term benefits from early nutritional treatments.[1],[2] Although algorithms differ, most CF NBS involve DNA analysis.[3] Diagnosis confirmation after a positive newborn screen is challenging as 2007 mutations have been identified to date in the CF Transmembrane Conductance Regulator (*CFTR*) gene (http://www.genet.sickkids.on.ca/cftr/Home.html), but their contribution to disease is undefined for most.[4, 5] The Clinical and Functional TRanslation of CFTR (CFTR2) project (www.CFTR2.org) characterizes disease liability focusing on mutations of high prevalence among CF patients. At the time of analysis, CFTR2 had characterized 202 mutations (178 as CF-causing, 12 as non CF-causing, and 12 as varying clinical consequence).[6] However, there remain over 1800 mutations of low frequency with uncertain disease liability.

California started CF NBS in July 2007.[7] To address the challenges of screening in an ethnically diverse state, a 3-step model was adopted that includes *CFTR* sequencing. Sequencing opened the opportunity to explore the full range of the CF spectrum by understanding the penetrance of *CFTR* mutations of varying and unknown clinical significance.

The aim of this study was to describe the phenotypes within a cohort of children with a positive California CF NBS carrying sequenced *CFTR* mutations categorized as varying clinical consequence or unknown disease liability by CFTR2. We previously reported data on children carrying one CF causing mutation in trans with one or more non CF-causing mutations.[8] The focus of the previous work was to test the new CFTR2 classification of *CFTR* mutations found to have low penetrance among families with children with CF, and therefore called non-CF causing. We showed that children detected by screening carrying one CF-causing and one or more non-CF causing mutation on the opposite chromosome had a benign phenotype in the first 2 to 6 years of life. Based on these findings, the California Department of Public Health, along with the California CF NBS consortium, recommended centers to label any screen-positive children (historical or new) carrying non-CF causing mutations as CF-related metabolic syndrome (CRMS); however with the caveat that in these cases they would be referred to CF centers for genetic counseling only and no further diagnostic testing or clinical follow up (policy implemented in July 2014). The focus of the present work is to address children carrying one CF causing mutation and one or more mutations of unknown clinical significance, which is particularly relevant to families with children with CRMS or CF screen positive, inconclusive diagnosis (CFSPID).”[9–12]

## Methods

### Study Design and Population

We conducted a retrospective cohort study of newborns with a positive California CF NBS from July 2007 to July 2011, with two to six years of follow up (data collected until August 2013). The California CF screening model has been described previously.[7] Briefly, it begins with an elevated immunoreactive trypsinogen (IRT; fixed cut off ≥62 ng/ml, 98.4th percentile), followed by a panel of 40 *CFTR* mutations previously identified as prevalent among CF patients in California.[13] Newborns with only one panel mutation had DNA from the original filter paper blood spot used for *CFTR* sequencing. Children with two or more mutations identified by the panel and/or sequencing were considered screen-positive and referred to a CF care center for diagnosis confirmation. Children with only one *CFTR* mutation were considered carriers and their parents offered telephone genetic counseling.

Sixteen CF centers in California received referrals from the NBS program and followed screen-positive children. As recommended by the Cystic Fibrosis Foundation (CFF) guidelines and the California Minimum Guidelines (http://www.cdph.ca.gov/programs/nbs), the first clinic visit occurred within a week after the CF NBS result was reported (~2–6 weeks of age), then again one month later, at 6 months, and at 12 months. After the first year, CF patients were seen quarterly and as clinically indicated; most subjects with CRMS were seen once or twice a year. [9, 14] A CRMS designation was given to children who: 1) had two *CFTR* mutations identified by screening, in which at least one was not classified as CF-causing by CFTR2; 2) were asymptomatic; and 3) had a sweat test < 60 mmol/L.[9] Clinical and laboratory data were entered into the state’s web-based screening information system for the initial assessment visits (within the first 6 to 12 months) until a diagnosis of CF or CRMS was established and yearly thereafter. After personal identifiers were removed, the data were sent to the Children’s Hospital Los Angeles (CHLA) research team for analysis. The CHLA Institutional Review Board and the California Health and Human Services Agency Committee for the Protection of Human Subjects approved the study and waived informed consent for participating families.

### *CFTR* sequencing

Gene scanning and sequence analysis was performed using Ambry Test®: CF (Aliso Viejo, CA, July 16, 2007—June 30, 2010) or direct *CFTR* DNA Sanger sequencing at Stanford Molecular Pathology Laboratory (July 1, 2010—June 30, 2011). Sequencing was performed by a semi-automated process described by Schrijver et al.[15] The assay covered 983 bases of 5’ untranslated region, 27 exons, 20 bases into the 5’ and 3’ ends of all introns, the *CFTR* poly T status and TG tract, intron 19 surrounding the 3849+10kbC>T mutation, and intron 11 surrounding the 1811+1634A>G mutation. Analysis for known and novel mutations in these areas of the gene was performed first by unidirectional sequencing, confirming all suspicious and positive mutations with sequencing in the opposite direction.

### Genotype Groups

In this work, we focus on two genotype groups: 1) the varying clinical consequence group (VCC) consisting of individuals with one CF-causing mutation from the California 40 panel and a second mutation identified by sequencing characterized as having varying clinical consequence by CFTR2, and 2) the unknown disease liability group (Unknown) consisting of those with one CF-causing mutation from the panel and a second mutation identified by sequencing characterized as unknown or not yet studied by CFTR2. A CF-causing group (CF-C) served as a reference group and consisted of individuals with two CF-causing mutations (one from the California 40 panel and the second either from the panel or from *CFTR* sequencing classified as CF-causing by CFTR2). Children who screened positive with one panel mutation and no additional mutation except a poly 5T in intron 8 by sequencing were excluded from the present study; they will be the focus of a separate article.

### Outcome Measures

In addition to genotype, study variables included characteristics used to make a clinical diagnosis of CF and to evaluate disease severity: sweat chloride (SC) concentration, pancreatic sufficiency status, growth parameters, rate of first acquisition of *Pseudomonas aeruginosa* in the first year of life, and persistent colonization with *P*. *aeruginosa*. Sweat tests were performed according to a standardized protocol and results categorized as positive (≥60mmol/L), intermediate (30–59mmol/L), or negative (<30mmol/L).[16] Those with initial negative or intermediate results had repeat testing at ~ 6, 12, and 24 months, and the highest value used in the analysis. Pancreatic status was assigned based on the most recent fecal elastase (FE) value as pancreatic insufficient (PI) if FE <200mcg/g or pancreatic sufficient (PS) if FE ≥200 mcg/g.[16],[17] In 20% of subjects missing FE values, pancreatic status was assigned based on use of pancreatic replacement enzymes (criteria also used by CFTR2 and the CFF patient registry).[6],[12] Growth analysis was examined in subjects with at least three growth measurements. As *P*. *aeruginosa* is a common and severity-defining pathogen in CF,[18] all subjects with at least one positive culture for this organism were reviewed separately with their respective centers to determine persistent colonization status. Cultures were obtained during clinic visits by deep-throat swabs as recommended by practice guidelines.[14],[9] Considering that first acquisition of *P*. *aeruginosa* may be transient and not pathognomonic of CF, we improved the disease-defining parameter by applying a modified Leeds criteria of persistent colonization.[19] Persistent colonization was determined as follows: a) mucoid-type, ever; and b) at least two positive cultures within twelve months. Other categories included: *P*. *aeruginosa*-yes-ever for subjects with positive cultures who did not meet the criteria for persistent; negative for those who never had a positive *P*. *aeruginosa* culture; and unknown for others with missing data (defined as fewer than two cultures per year and/or fewer than two years of follow up). Lost to follow up was defined as no medical visit for ≥ eighteen months.

### Statistical analysis

Comparisons among the 3 genotype groups on demographic and phenotype characteristics were made with analysis of variance or Wilcoxon rank sum tests for continuous variables and Chi-square tests for categorical variables. When statistical significance was observed among the groups, Bonferroni-adjusted pairwise comparisons were performed. Longitudinal weight-for-height z-scores (based on CDC growth curves from year 2000) during the first 12 months of life were analyzed using quadratic repeated measures mixed models, with CF-C further divided into PI and PS. Outlier growth data points were removed if not biologically plausible. Dunnett’s *post hoc* comparisons of least squares means were made with CF-C-PI children as the reference group. Time to first positive culture for *P*. *aeruginosa* during the first year of life was analyzed with the Kaplan-Meier method and compared between groups with the log-rank test. Despite gender differences across groups, multivariate analyses on continuous variables found no significant interaction by gender; therefore, unadjusted results are reported. Statistical analysis was performed with SAS/STAT® v9.2 software. Statistical tests were 2-sided with a Type I error of p<0.05.

## Results

**Fig 1:**
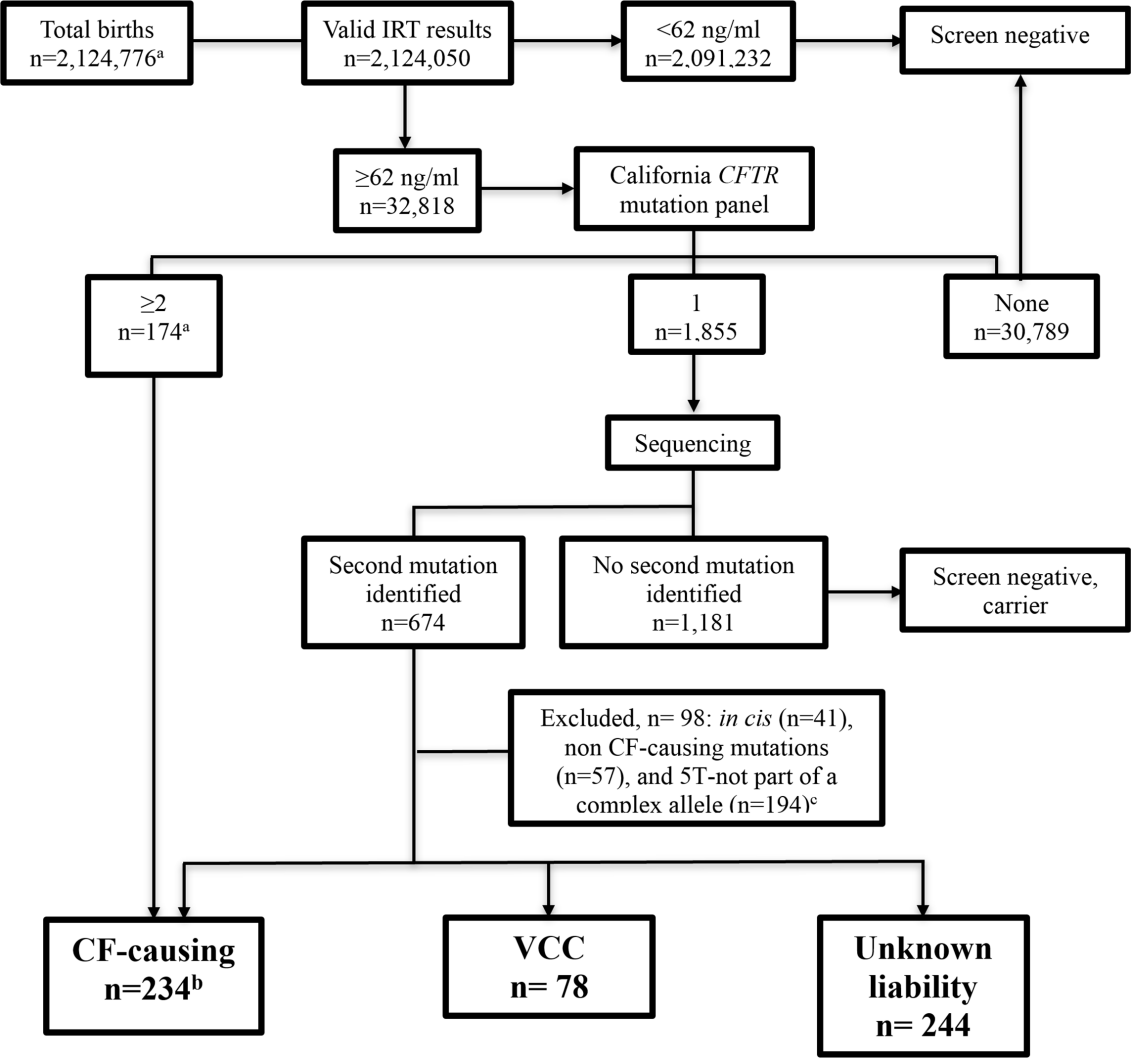
Flowchart. ^a^Period July 16, 2007 to July 31, 2011 (total births from July 1, 2007 to July 31, 2011 as originally described in the non CF-causing cohort publication was 2,178,829).^8^ IRT = immunoreactive trypsinogen. ^b^CF-causing = group of children carrying *CFTR* mutations from the panel *in trans* with another panel mutation (n = 174) or with a sequenced mutation classified as CF-causing by CFTR2—July 2013 list (n = 60). Total of subjects in the CF-C group in previous publication of non CF-causing cohort was 226. [8] The difference is due to changes in CFTR2 classification from 2012 to 2013. VCC = group of children carrying *CFTR* mutations from the panel *in trans* with a sequenced mutation of varying clinical consequence. Unknown liability = group of children carrying *CFTR* mutations from the panel *in trans* with a sequenced mutation of unknown disease liability. ^c^ 5T = group of children carrying *CFTR* mutations from the panel *in trans* with / IVS8-(TG)m-5T subgroups (TG-13, 12, and 11) will be reported elsewhere.

Among 2,124,776 births during the 4-year study period, 32,818 had further testing due to IRT levels above the cutoff of 62 ng/ml. After exclusion of those with mutations *in cis*, non CF-causing mutations, and intron 8 variations (poly-T and TG tract), three study groups were formed: CF-C, VCC, and Unknown (Fig 1).

The groups differed significantly on IRT (p<0.0001), birth weight (p = 0.0005) and meconium ileus (p<0.0001), with lower median IRT, higher mean birth weight, and no cases of meconium ileus among VCC and Unknown subjects compared to the CF-C group (Table 1). Individuals in the VCC and Unknown groups were more likely to be female (p = 0.0007), compared to the CF-C group. Fifty-four percent of those in the Unknown and 37% in the VCC groups were lost to follow up, compared to 18% in the CF-C group (p<0.0001).

**Table 1:** Description of the Study Population and phenotype characteristics.

Population Characteristics	CF-C^a^ (n = 234)	VCC^b^(n = 78)	Unknown^c^ (n = 244)	p value^d^
Age at last follow up, mean±SD, months	49±14	48 ±13	49±13	0.91
Female gender, n (%)	99 (42.3)	45 (57.7)	144 (59.0)	0.0007
Birth weight, mean±SD, kg	3.11±0.61	3.34±0.54	3.30±0.58	0.0005
IRT, median (IQR)^e^, ng/mL	162 (109,235)	89 (74,116)	80 (69,106)	<0.0001
Meconium Ileus, n (%)	36 (15.4)	0 (0)	0 (0)	<0.0001
Pancreatic Insufficiency, n (%)	181/228 (79)	3/75 (4)	16/208 (8)	<0.0001
Lost to follow up, n (%)	42 (18)	29 (37)	131 (54)	<0.0001
Race/ethnicity, n (%)				
Whites	113 (48)	29 (37)	87 (36)	
Hispanics	93 (40)	40 (51)	110 (45)	0.17^**f**^
Non-Hispanics Blacks	12 (5)	2 (3)	17 (7)	
Multiple and others	16 (7)	7 (9)	30 (12)	
Maximum Sweat Chloride, median (IQR), mmol/L ^g^	94 (83,103)	30 (20, 40)	18.5 (12, 30)	<0.0001
Sweat Chloride distribution, n (%), mmol/L				
<30	4 (2)	37 (48)	168 (74)	<0.0001
30–59	21 (10)	39 (51)	33 (14)	<0.0001
≥ 60	190 (88)	1 (1)	27 (12)	<0.0001
*P*. *aeruginosa* during 1^st^ year, Probability (s.e.) ^h^	0.18 (0.03)	0.11 (0.04)	0.08 (0.02)	0.0088
*P*. *aeruginosa* colonization, n (%)				
Unknown	40 (17)	41 (52)	150 (61)	n/a
Never	90 (38)	23 (30)	57 (24)	n/a
Yes-ever	53 (23)	7 (9)	23 (9)	n/a
Persistent	51 (22)	7 (9)	14 (6)	n/a

^a,b,c^ Denominators are the total of subjects per group unless otherwise specified.

^a^ Two CF-causing mutations (one from the California 40 panel plus one sequenced and classified as CF-causing by CFTR2).

^b,c^ Children identified with one CF-causing mutation from the California 40 panel plus one or more sequenced classified as varying clinical consequence ^b^ or unknown disease liability. ^c^ Unknown disease liability includes the 6 mutations in CFTR2 classified as unknown as well as those not yet studied (CFTR2 list from July 2013).

^d^ p values indicate the comparison of all 3 groups. If p<0.05 there is a difference between at least 2 groups. Pairwise difference noted in the text.

^e^ IRT = Immunoreactive Trypsinogen.

^f^ p value for Hispanics compared to all other races and ethnicities.

^g^ Sample sizes are as follows: CF-C = 215, VCC = 77, and Unknown = 228

^h^ Sample sizes are as follows: CF-C = 231, VCC = 78, and Unknown = 243

In the study population, initial sweat tests were performed at a median age of 54 days (interquartile range [IQR]: 41–75) days. Testing was repeated at least once in 70% of those with an initial result <60 mmol/L or inadequate quantity of sweat. One subject (1%) from the VCC group (genotype: F508del *in trans* with R117H/7T) and 27 (12%) from the Unknown group met SC diagnostic criteria for CF (≥60 mmol/L) (Table 1). The genotypes among those with diagnostic SC levels and unknown liability mutations are shown in Table 2. During the study period, the SC conversion from <60 mmol/L (indeterminate) to ≥60 mmol/L (diagnostic of CF) happened in 9 (4%) subjects from the CF-C group, 1 (1.3%) from the VCC (genotype: F508del *in trans* with R117H/7T); and 4 (1.6%) from the Unknown group (genotypes: F508del *in trans* with L32M, S1159P, or T1076P [n = 3] and 663delT *in trans* with I105N [n = 1]). The mean (±SD) conversion age was 16±11 months.

**Table 2:** Subjects carrying one CF-causing from the California 40 panel plus one or more sequenced mutations of unknown disease liability who met diagnostic criteria for CF based on sweat chloride levels and/or pancreatic insufficiency (PI) status. Mutations are described by legacy names.

Unknown disease liability group: Individual genotypes				
n	CF-C mutation	Sequenced mutation 1	Sequenced mutation 2	Annotation of sequenced mutations 1and 2
**Sweat chloride 30–59 mmol/L and PI**				
1	F508del	2789+2insA	-	Non-canonical splice
**Sweat Chloride ≥60 mmol/L and PI**				
1	F508del	1138insG	-	Frameshift
1	F508del	F1016S	L102R	Missense, missense
1	F508del	1343delG	-	Frameshift
1	F508del	296+28A>G and 2686-2687insT	-	Non-canonical splice and frameshift
1	F508del	2481_2482insT	-	Frameshift
2	F508del	2215insG	D836Y	Frameshift, missense
1	F508del	I1005R	-	Missense
1	F508del	3199del6	-	In-frame deletion
1	F508del	-816C>T	F1107L	Promoter, missense
1	F508del	1410delC	I556V	Frameshift, missense
1	F508del	3015_3018dupGTCA	-	Frameshift
1	S549N	1949del84	-	In-frame deletion
1	W1089X	1811+1G>A	-	Canonical splice
1	R75X	T1036N	-	Missense
**Sweat Chloride ≥60 mmol/L and PS**				
1	F508del	S1159P	-	Missense
1	F508del	T1076P		Missense
1	F508del	L32M	-	Missense
1	F508del	T1036N	-	Missense
1	F508del	c.-152G>C*	-	Promoter
1	F508del	G126D	-	Missense
1	F508del	Y917C	-	Missense
1	P205S	K114del	-	In-frame deletion
1	N1303K	K162E	-	Missense
1	N1303K	Q359K/T360K	-	Missense
1	663delT	I105N	-	Missense
1	935delA	T1036N	-	Missense

* Only cDNA name available for this mutation.

Pancreatic insufficiency was observed in 4% of the VCC and 7.7% of the Unknown groups, compared to 79% of the CF-C group (p<0.0001; Table 1). Pancreatic insufficiency in the VCC group occurred in individuals carrying F508del *in trans* with the following mutations: R1070W (n = 1: FE = 153 μg/g at 3 months), D1152H (n = 1: FE converted from 244 μg/g at 2 months to 144 μg/g at 8 months), and R117H/7T (n = 1: FE converted from 451 μg/g at 6 months to 155 μg/g at 28 months). Genotypes of those with PI and/or SC ≥60mmol/L in the Unknown group are described in Table 2. Conversion from PS to PI was seen in 4 subjects: one (0.4%) from the CF-C group (homozygous 663delT), two (2.6%) from the VCC group (mentioned above) and one (0.4%) from the Unknown group (genotype: S549N *in trans* with 1949del84; FE converted from 301 μg/g at 2 months to <15 μg/g at 20 months), setting the conversion rate at 1.3% of subjects initially labeled PS. The mean (±SD) age of conversion was 15±11 months. There were 4 subjects whose initial FE values were consistent with PI (between 50 and <200 mcg/g), but who later converted to PS (>200 mcg/g).

**Fig 2:**
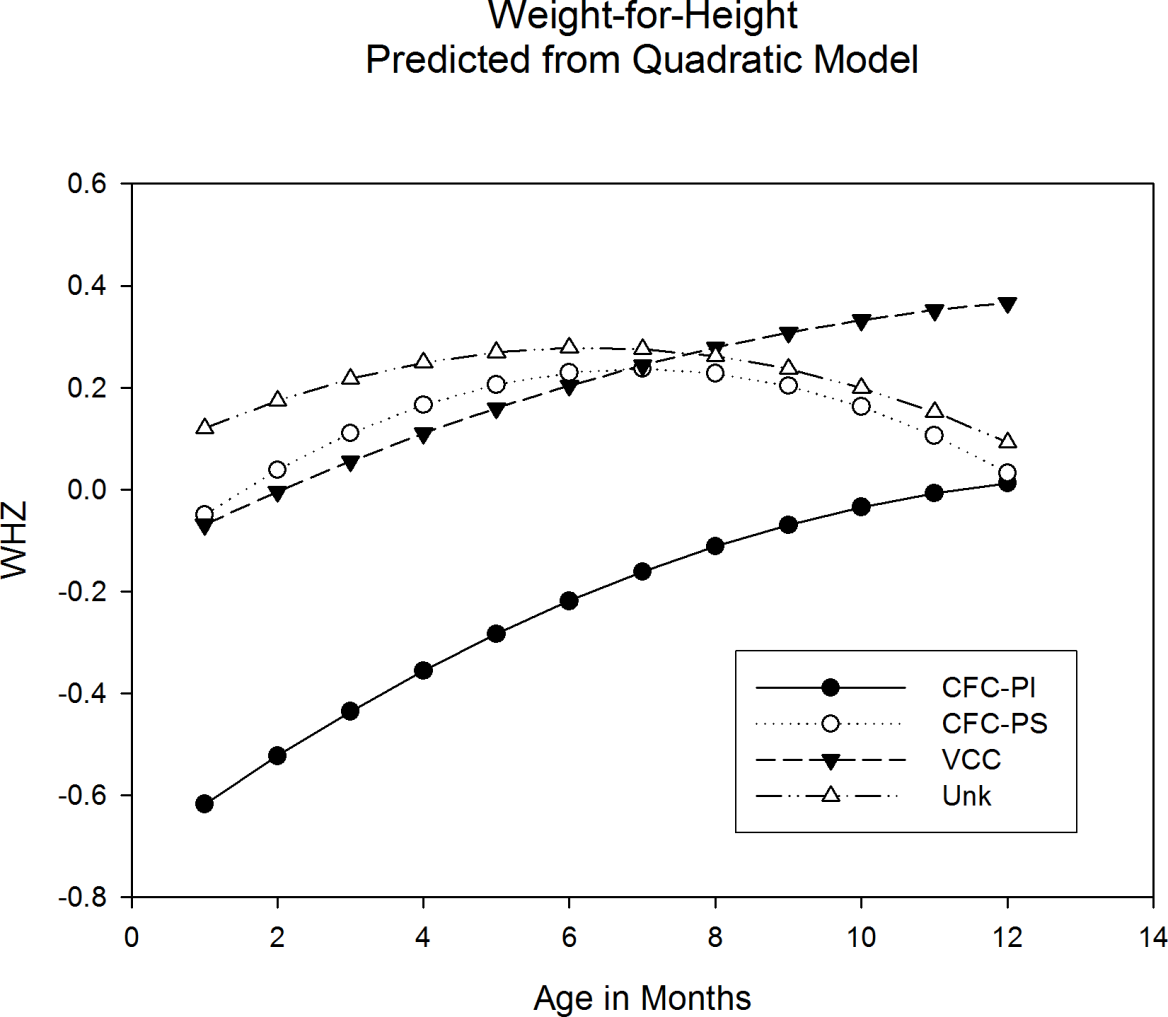
Weight for height over the first twelve months of life. Study subgroups: CF-C-PI = CF-causing group, pancreatic insufficient; CF-C-PS = CF-causing group, pancreatic sufficient; VCC = mutations of varying clinical consequence group; and Unk = mutation of unknown liability group. Pancreatic insufficient subjects were removed from VCC (n = 3) and Unk (n = 16) groups for this analysis. There was a statistically significant difference between CF-C-PI and CF-C-PS (p = 0.0247), VCC (p = 0.0013), and Unk (p<0.0001). There is no statistically significance difference between CF-C-PS and VCC and Unk (respectively p = 0.9767 and p = 0.9922). Pairwise comparisons indicate a significant difference of WHZ between CF-C-PI and CF-C-PS, VCC, and Unk at 1m (p<0.0001) and at 6 months (p = 0.0003), and no statistically significant difference at 12 months (p = 0.177), suggesting nutritional recovery in the CF-C-PI group.

Repeated measures analyses of weight-for-height z-scores (WHZ) through 12 months were conducted for 492 subjects, with CF-C grouped by PS/PI status and excluding PI subjects from the VCC and Unknown groups due to small sample sizes. PS subjects in all groups had significantly better growth compared to CF-C-PI subjects (Fig 2). Evaluation of WHZ at specific time points indicate significant differences among groups at 1 month and at 6 months, with CF-C-PI lower than other groups at both ages, but no significant differences at 12 months, suggesting nutritional recovery in the CF-C-PI group.

**Fig 3:**
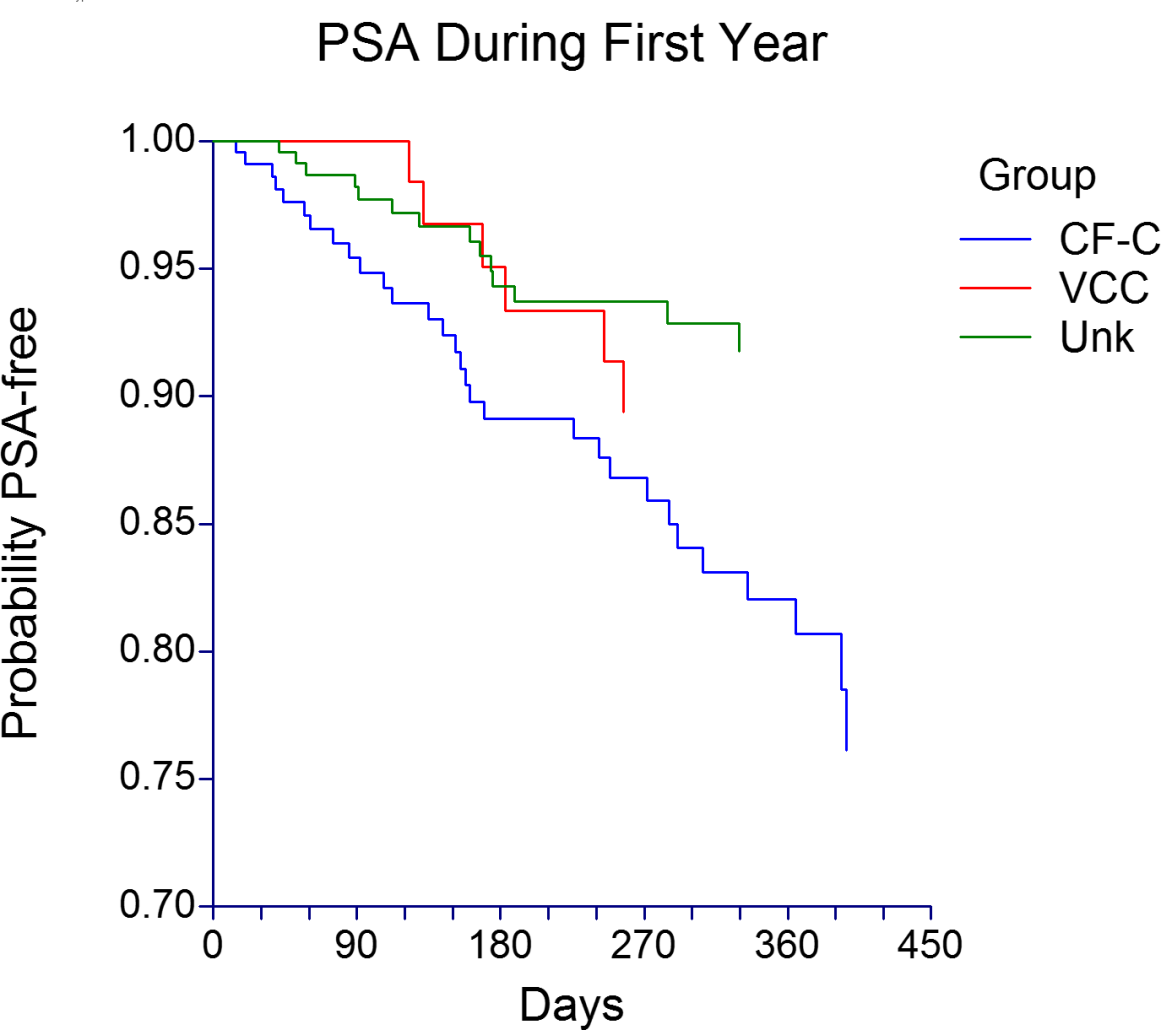
*Pseudomonas aeruginosa* first acquisition in the first 12 months of life. PSA = *Pseudomonas aeruginosa*, CF-C = CF-causing group (n = 231), VCC = mutation of varying clinical consequence group (n = 78), Unk = mutation of unknown liability group (n = 243). The probability of acquiring PSA was different among the 3 groups (p = 0.0088 Log-Rank test). CF-C group had a probability of acquisition rate of 0.18 (s.e. 0.03), which was not statistically significantly different than the VCC group (0.11 s.e. 0.04, p = 0.0916), but statistically higher than the Unk group (0.08 s.e. 0.02, p = 0.0034) according to pairwise comparisons.

In the first 12 months of life *P*. *aeruginosa*-free probability was lowest in CF-C subjects. Likewise, the probability of acquiring *P*. *aeruginosa* differed significantly among the 3 groups, higher in the CF-C group compared with the Unknown group, but not statistically different when compared with the VCC group (Fig 3). For a large proportion of subjects in VCC and Unknown groups, persistent colonization status was not determined due to missing data (52% and 61% respectively), so no statistical tests were performed. Among those with sufficient data, 9% in VCC and 6% in Unknown groups met criteria for persistent colonization (Table 1).

Next, we sought to identify prevalent mutations that could be characterized as benign or deleterious in the VCC and Unknown groups (Table 3). For mutations observed ≥6 times, SC levels were below the diagnostic threshold in the VCC and Unknown groups except for one subject (genotype: F508del *in trans* with R117H/7T) whose SC reached 63 mmol/L. A high percentage of subjects with D1152H and R117H/7T (70% and 64%, respectively) had SCs between 30 and 59 mmol/L, suggesting some CFTR dysfunction, though only one subject with each mutation was PI (4% of total VCC group). Persistent *P*. *aeruginosa* colonization was observed in 5/24 (21%) and 1/17 (6%) of the VCC and Unknown groups, respectively. Among the frequent unknown liability mutations, 1525-42G>A, L320V, L967S, R170H, and 296+28A>G, when not part of a complex allele and when in combination with a CF-C mutation, had SC results ≤ 33 mmol/L, no cases of pancreatic insufficiency, and only one isolated case of persistent *P*. *aeruginosa* (Table 3).

**Table 3:** Subjects with one CF-causing mutation from the California 40 panel plus a second mutation identified by sequencing and classified as varying clinical consequence or unknown disease liability, with frequency ≥ 6.

Legacy name	n	Highest Sweat Chloride in mmol/L: Median, range *(total included)*	PI: n/total included	PSA–P: n/total included
**Sequenced *CFTR* mutations—varying clinical consequence**				
D1152H	23	32,16–56 *(23)*	1/21	3/14
R117H/7T	22	32,17–63 *(22)*	1/21	2/10
F1052V	11	18,11–54 *(10)*	0/11	0/5
**Sequenced *CFTR* mutations—unknown disease liability**				
1525-42G>A	19	12, 8–33 *(19)*	0/19	1/6
L320V	13	14, 10–26 *(12)*	0/12	0/4
L967S	9	20, 12–27 *(7)*	0/6	0/2
R170H	9	20, 7–30 *(8)*	0 /6	0/3
296+28A>G	6	14, 9–23 *(6)*	0 /4	0/2

PI = pancreatic insufficiency.

PSA-P = *Pseudomonas aeruginosa* persistent colonization according to a modified Leeds criteria.

## Discussion

The California CF NBS protocol uses DNA sequencing to identify a second *CFTR* mutation in individuals with elevated IRT and one commonly seen CF-causing mutation. Within a larger analysis aimed at describing the genotype and phenotype variability in this ethnically diverse population, the current study demonstrates that most infants with a positive CF NBS with *CFTR* mutations of VCC or Unknown disease liability do not meet the diagnostic criteria for CF in early childhood. Children with a CF-C genotype had classical disease phenotype (88% had SC ≥60 mmol/L, 79% were PI, 18% acquired *P*. *aeruginosa* in the first year, and 22% met criteria for persistent colonization), while those in the VCC and Unknown groups had greater phenotypic variability (respectively, 1.3% and 12% had SC ≥60 mmol/L, 4% and 8% were PI, 11% and 8% acquired *P*. *aeruginosa* in the first year, and 9% and 6% met criteria for persistent colonization). However, 5% of VCC and 11% of Unknown group subjects met diagnostic criteria of CF by SC ≥60 mmol/L and/or pancreatic insufficiency status.

NBS protocols using DNA analysis will identify individuals who screen positive but on initial evaluation may not have CF. The population studied here is unique in that it allows analysis of both grouped and individual genotypes to determine penetrance (number of individuals that will develop CF out of total having the genotype). Of 556 subjects studied, there were 290 subjects (52%) with *CFTR* mutations identified who did not meet CF diagnostic criteria. A portion of these patients may never have disease; while others will go on to develop CF or CFTR-related disorder (CFTR-RD).[20, 21] Perhaps most at risk are those with intermediate SC values (51% of VCC and 14% of Unknown). As these individuals age and develop symptoms, we will have a better understanding of their mutation’s penetrance. They may progress to a CF phenotype independent of SC results; nearly 14% of individuals diagnosed with CF as adults in the CFF patient registry had sweat values <60 mmol/L, with the CF diagnosis established by genotype or clearly recognized phenotype.[16]

Specific phenotypes, independent of genotype, may be useful to predict the development of disease. Pancreatic status predicted growth pattern, suggesting that clinicians may rely on fecal elastase measurements to assess risk of malnutrition across all genotypes. Our analysis suggests that PI subjects recovered nutritionally in the first 12 months. As suggested by Farrell *et al*, this is a measure of success of a CF-NBS program, as it reflects early and proper nutritional management for those at high risk of malnutrition.[22] As previously seen, there was fluctuation in the levels of FE.[17] Conversion from PI to PS (n = 4) and PS to PI (n = 4) occurred beyond the first 3 months of life in children from our study. Therefore, if the initial FE value is equivocal, repeat FE at 12 months should be considered.

*P*. *aeruginosa* colonization in subjects with mutations of varying and unknown significance (11% and 8% respectively) was higher than reported for healthy controls, lower than classical CF, and similar to previous reports on children with CRMS. The presence of *P*. *aeruginosa* is considered pathognomonic for CF and represents an important contributor to lung function decline.[18, 23] The reported incidence of first acquisition in the first year of life in CF patients is approximately 25%,[24] compared to 1–3% in healthy controls[25], [26] and overall 11% in CRMS.[12] In our study, CFTR dysfunction among VCC and Unknown subjects could explain the higher incidence of *P*. *aeruginosa*. However, the increased exposure to other CF patients and frequent surveillance are other possible explanations, which raises the concern of how the high detection rate may influence the final diagnosis of CF. [24],[27] Alternatively, these children may have transient colonization similar to normal controls.

Both classical CF and benign phenotypes were seen with VCC and Unknown genotypes. Though the majority of subjects identified in VCC and Unknown groups had a benign presentation, the full spectrum of phenotype associated with *CFTR* allelic heterogeneity was observed. Ten percent (32/322) in the VCC and Unknown groups combined met either SC or PI criteria for CF diagnosis after screening. Interestingly, several subjects from the Unknown group who had a final CF diagnosis (Table 2) had 3 *CFTR* mutations, bringing attention to our limited understanding about complex alleles. This is not so problematic for patients who have a clear diagnosis of CF due to early positive phenotype; however, individuals carrying mutations of unknown significance and who have a negative phenotype in the early years will not know their risk of developing disease unless phasing is done and their families are willing to continue to follow and monitor for early symptoms.

Consistent with previously published reports, there was a high prevalence of subjects with one CF-causing mutation and a second mutation known to be associated with variable penetrance for CF and CFTR-RD such as D1152H and R117H.[28–30] A study of CF patients with one copy of D1152H and another CF-causing mutation has shown low incidence of symptoms in childhood, but up to 70% of adults have bronchiectasis.[29] Though this may be biased towards severe cases referred to CF centers, it illustrates the potential of these mutations to produce a pathologic phenotype that may be able to be averted. Children identified in this study may better represent the spectrum of penetrance of these genotypes, as the SC values are lower than published reports of CF patient registry data.[6]

Our study has also brought attention to mutations appearing less likely to cause CF. 1525-42G>A, an intronic mutation rarely described in the literature[31], was present in 19 children in this cohort. All had a negative SC and were PS. Its frequency of 0.4% in the general population and 2.2% in Hispanics (www.1000genome.org) explains the high prevalence in our population, suggesting that this is a benign polymorphism. Other frequently identified mutations with benign presentations included L320V, L967S, and R170H. These are rare missense mutations described in case reports of individuals with negative SC and CBAVD or pancreatitis, but are not known to cause CF[32–34].

The strength of our study is the large cohort of children from an ethnically diverse population who underwent *CFTR* DNA sequencing as part of NBS and the centralized database available to input clinical and laboratory information. There are two main limitations: relatively short follow up period and high number of subjects lost to follow up. The full penetrance of some VCC and Unknown mutations that may result in CF or CFTR-RD will not be apparent until adulthood.[21] Thus, we would need a longer follow up to describe each mutation’s penetrance. Despite repeated efforts by the NBS program to ensure thorough reporting of all positive-screen cases by CF centers, follow-up of children was less complete in the VCC and Unknown groups than for the CF-C group, especially after one year of age. Because children becoming symptomatic will presumably return to a CF center for care, differential underreporting of CF by the groups was probably small.

This study helps us understand the penetrance of *CFTR* mutations and the full spectrum of CFTR-related disease that may begin in early childhood. Although *CFTR* sequencing is not done from the same dry-blood spot as the IRT measurement by other CF NBS models, it is a technology available for all and used post-referral in most screen-positives with one mutation detected and intermediate SC results.[35] These data can help clinicians, investigators, public health experts, and parents understand the risk of CF for children with mutations of uncertain clinical significance and impact clinical care protocols. An ultimate goal of this work was to identify mutations that are prevalent and benign (i.e.1525-42G>A), so that these infants will not be considered at risk to develop CF. This process will avoid over-medicalization of individuals at low risk to develop early disease and increase the positive predictive value of this screening program. Similarly, identifying mutations that are deleterious (i.e., 2215insG with D836Y) will avoid losing these high-risk infants from CF center follow-up. More reliable *in vivo* CFTR function assays or other discriminating biomarkers are needed to address the issue of uncertain prognosis in CRMS subjects. Long-term follow up of this cohort is required to better understand how *CFTR* mutations and other genes contribute to disease beyond our current understanding of classical CF.
